# Clinical Outcomes for Diabetic Foot Ulcers Treated with Clostridial Collagenase Ointment or with a Product Containing Silver

**DOI:** 10.1089/wound.2018.0784

**Published:** 2018-10-11

**Authors:** Travis A. Motley, Joseph M. Caporusso, Darrell L. Lange, Robert A. Eichelkraut, David Innes Cargill, Jaime E. Dickerson

**Affiliations:** ^1^ Department of Orthopedics, John Peter Smith Hospital and Acclaim Physician Group, Fort Worth, Texas.; ^2^ Complete Family Footcare, McAllen, Texas.; ^3^ Smith & Nephew, Fort Worth, Texas.; ^4^ Graduate School of Biomedical Sciences, University of North Texas Health Science Center, Fort Worth, Texas.

**Keywords:** diabetic foot ulcer, collagenase, silver, debridement, infection, wound healing

## Abstract

**Objective:** To compare outcomes of diabetic foot ulcers (DFUs) treated with clostridial collagenase ointment (CCO) or silver-containing products, both in combination with sharp debridement as needed.

**Approach:** One hundred two subjects with qualifying DFUs were randomized to daily treatment with either CCO or a silver-containing product for 6 weeks followed by a 4 -week follow-up period. The primary outcome was the mean percent reduction in DFU area. A secondary outcome was the incidence of ulcer infections between groups.

**Results:** At the end of treatment, the mean percent reduction in area from baseline of DFUs treated with CCO was 62% (*p* < 0.0001) and with silver was 40% (*p* < 0.0001). The difference between groups—22%—was not statistically significant (*p* = 0.071). Among ulcers closed by the end of treatment, the mean time to closure was 31.1 ± 9.0 days versus 37.1 ± 7.7 days, respectively (not statistically significant). There was a numerically greater incidence of target ulcer infections in the silver group (11, 21.6%) than in the CCO group (5, 9.8%; *p* = 0.208). No clinically relevant safety signals were identified in either group.

**Innovation:** CCO treatment can progress a wound toward closure. Ulcer infection prophylaxis may not be sacrificed when treating DFU with CCO in lieu of silver-containing products.

**Conclusion:** Both CCO and silver-containing products promote significant reduction in DFU area over 6 weeks of treatment with no clinically relevant safety concerns. Mean percent reduction in lesion area was numerically (22%) but not significantly greater with CCO compared to silver, as was time to ulcer closure, with an incidence of ulcer infection at least as low as for silver-containing products.

**Figure:**
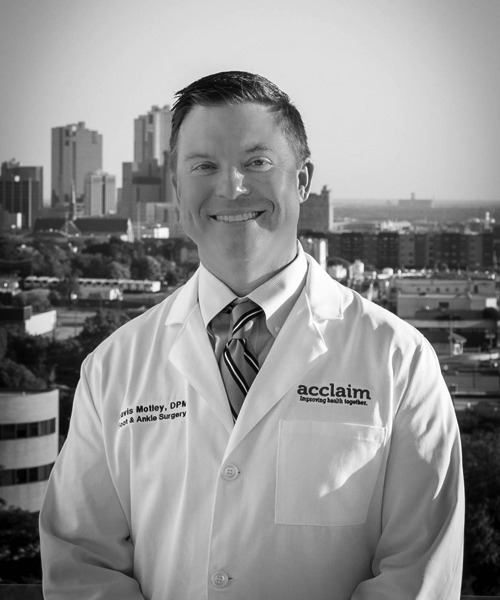
**Travis A. Motley, DPM, MS, FACFAS**

## Introduction

There are more than 22 million people in the United States with diabetes mellitus,^1^ of whom up to 25% are at risk for the development of diabetic foot ulcers (DFUs).^2^ In the United States, the annual cost of caring for a patient with a DFU ranges from ∼$12,000–$16,000.^3^ Cumulatively, and including the cost of infection, Medicare spending in 2014 for DFU is estimated at nearly 19 billion USD.^4^ In Canada, the annual costs associated with DFU care exceeded $500 million Canadian dollars (CAN), with an average 3-year cumulative cost of greater than $50,000 CAN per incident case.^5^

Open DFUs are typically heavily colonized by bacteria and consequently at risk of becoming infected.^6^ Such infections, if not adequately treated, can progress to sepsis or result in amputation.

It is generally accepted that there are five tenets of good DFU care, including glucose control, maintenance of a moist wound environment, debridement, offloading, and infection prevention/management. To assist with healing, diabetic patients should strive to keep glucose levels as close to normal as possible, using a combination of proper diet and glucose-lowering medications. The ulcer should be kept moist, but not wet, during the healing process. Removal of dead or necrotic tissue can be accomplished via periodic sharp debridement with or without ongoing enzymatic debridement. Offloading is essential to reduce or eliminate pressure on the wound. Infection prevention and management are also important. Frank infections warrant antibiotic therapy; prophylaxis is often accomplished using a silver-containing product. Silver is a broad-spectrum antimicrobial, and its use in DFU has become more common in recent years. In a recent clinical trial evaluating the role of clostridial collagenase in DFU treatment, the control group was managed via Investigators' preferred standard of care, which included a silver-containing product in 63% of cases.^7^ One potential limitation of silver use in DFUs is that silver ions are cytotoxic to human dermal fibroblasts and epithelial keratinocytes,^8^ such that infection prevention may come at a cost of slowed wound healing, depending on the exposure level to silver ions.^9,10^

Ongoing enzymatic debridement can be accomplished using an ointment containing collagenase derived from the bacterium *Clostridium histolyticum* (Santyl^®^; Smith & Nephew, Fort Worth, TX). This proteinase specifically degrades collagen types I–V, and is currently the only product in its class available in the US marketplace. Previous laboratory studies have demonstrated that enzymatic debridement with clostridial collagenase promotes key aspects of wound healing, including granulation tissue formation and reepithelialization better than nonspecific proteases or inactive controls^11^; and may suppress inflammatory markers while promoting markers of inflammation resolution.^12^ Multiple clinical studies have demonstrated that when paired with sharp debridement, clostridial collagenase results in more rapid reduction in ulcer area and shorter time to closure than standard therapies.^6,7,13^

Optimal comprehensive DFU therapy would both promote progress toward closure and prevent infection. Comanagement with clostridial collagenase and silver-containing compounds has to be carefully managed, as the type and amount of silver may affect collagenase activity.^14^ However, findings from two recent studies suggest that infection rates may not be different among DFUs treated with clostridial collagenase or silver-containing products.^6,7^

## Clinical Problem Addressed

The current study compares wound progression toward closure in DFUs treated with either clostridial collagenase or silver-containing products, with the primary outcome being the mean percent reduction in DFU area and the secondary outcome being the incidence of ulcer infections in the two groups.

## Materials and Methods

This was a phase 4, prospective, randomized parallel-group open-label study conducted at 14 sites in the United States and Canada in accordance with the tenets of Declaration of Helsinki. The protocol was approved by all relevant ethics committees and all participants provided written informed consent.

Subjects were age 18 years or older with diabetes mellitus Type 1 or 2 requiring insulin or oral medications to control blood glucose levels and who had at least one qualifying DFU. Qualifying lesions were present on any part of the plantar surface of the neuropathic foot or hallux at least 5 cm from any other DFUs, measured 0.5–10 cm^2^ inclusive, were of at least 6 weeks and no more than 52 weeks duration, manifested no clinical signs or symptoms of infection, and required debridement. While there was no run-in phase, ulcers decreasing below 0.5 cm^2^ or increasing above 12 cm^2^ between Screening and Baseline visits were excluded. Adequate arterial perfusion (ankle brachial index >0.70 and ≤1.20 at or near the ulcer site) was required. A complete list of eligibility criteria is given in the Appendix 1.

The study period consisted of three phases: randomization/baseline, treatment, and follow-up. At baseline, the target ulcer was cleaned with sterile saline, patted dry, and underwent sharp debridement as medically warranted and then photographed and measured using the ARANZ Silhouette ulcer imaging and measuring device (ARANZ Medical, Christchurch, New Zealand). Both the lesion area and perimeter were recorded. A 4 mm punch biopsy was obtained for quantitative bacteriology assessment. Qualifying subjects were then randomized in a 1:1 ratio into two treatment groups stratified by ulcer size (0.5–2.0 cm^2^ vs. >2.0 cm^2^) and ulcer duration (6–12 weeks vs. 13–52 weeks). The collagenase treatment group received clostridial collagenase ointment (CCO, Santyl; Smith & Nephew) applied once daily in a nickel-thick layer to the ulcer bed, covered in successive in-to-out layers with a nonadherent dressing (Allevyn; Smith & Nephew), a single layer of cast padding, and a self-adherent wrap bandage. The control group received treatment with a silver-containing product of the Investigator's choosing, used as indicated in the products' labels. Subjects in both groups were instructed to replicate their assigned treatment daily (or as appropriate for the Investigator selected dressing) throughout the treatment period. All subjects in both groups were provided with an appropriate offloading device (Darco shoe with PegAssist insole, Darco International, Huntington, WV), trained in its use, and instructed to wear the device whenever ambulatory.

During the 6-week treatment period, participants were examined weekly and their interim history and adverse events recorded. The target ulcer was cleaned and inspected, debrided at the Investigator's discretion, swabbed for bacteriology if deemed infected, and measured/recorded with the ARANZ device. If the target lesion was closed (100% reepithelialization with no drainage and no need for dressing), the subject immediately entered the 4-week follow-up phase. If not closed, the randomized treatment was continued and weekly follow-up maintained for up to 6 weeks. Use of the offloading device was reinforced at every visit.

Participants reaching the Week 6 visit (and those whose target ulcers achieved closure before Week 6) entered the follow-up phase of the study. During this 4-week period, ongoing ulcer treatment was entirely at the discretion of the Investigator. Participants were examined 2 and 4 weeks after entering the follow-up period, at which time adverse events were solicited and the target ulcer was assessed as closed, not closed, or reopened. Study exit occurred at the completion of the 4-week follow-up visit.

The primary efficacy outcome measure for this study was the mean percent change from baseline in the target ulcer area over the 6-week treatment period. A two-way analysis of covariance (ANCOVA) on the intent-to-treat (ITT) population was utilized to assess between-group differences. Secondary outcomes included the mean percent change in target ulcer area at the end of the follow-up period using the same ANCOVA model. The within-group change in ulcer area (both absolute and percent) from baseline to end of treatment was evaluated using a one-sample *t*-test. The number of target ulcer infections between groups was compared using analysis of variance (ANOVA), and the proportion of subjects in each group with target ulcer infections was compared using the Cochran–Mantel–Haenszel (CMH) test. Exploratory outcomes included the time to ulcer closure (in days) using Kaplan–Meier survival analysis, the proportion of ulcers closed using the CMH test, and the effect of bacterial burden on ulcer closure using logistic modeling. A *post hoc* analysis analyzed the percentage reduction in target ulcer area using a stepwise regression method that considered treatment and all baseline characteristics for the model. At a minimum, the model contained treatment and pooled site and further variables were added to the model at each step using a forward selection process. The final model included the terms for treatment, pooled sites, target ulcer age, ankle-brachial index (ABI), and body mass index (BMI). Safety assessment included descriptive statistics of adverse events using Medical Dictionary for Regulatory Activities coding, with severity (mild/moderate/severe) and treatment-relatedness (not/possibly related) assessed for each adverse event.

Sample size determination was informed by observations in previous studies of an ∼40% reduction in mean ulcer area over the planned study duration and a collagenase treatment effect size of 0.3–0.8. Assuming an effect size of 0.6 for CCO, 45 subjects per group would provide 80% power to detect this effect size at a 2-sided alpha level of 0.05 using a two-sample *t*-test. A sample size of 100 subjects was planned to account for attrition. The primary efficacy analysis was conducted in the ITT population (all randomized subjects with valid baseline ulcer measurements). Secondary and exploratory endpoints were conducted in both the ITT and per protocol (PP) populations (the PP population included all subjects meeting eligibility criteria and followed through the shorter of the 6-week treatment period or until ulcer closure with no significant protocol deviations). Safety analyses were conducted in the safety population (all randomized subjects receiving at least one treatment with study medication). Imputation for missing data was achieved using the last observation carried forward method.

## Results

**Figure 1.**
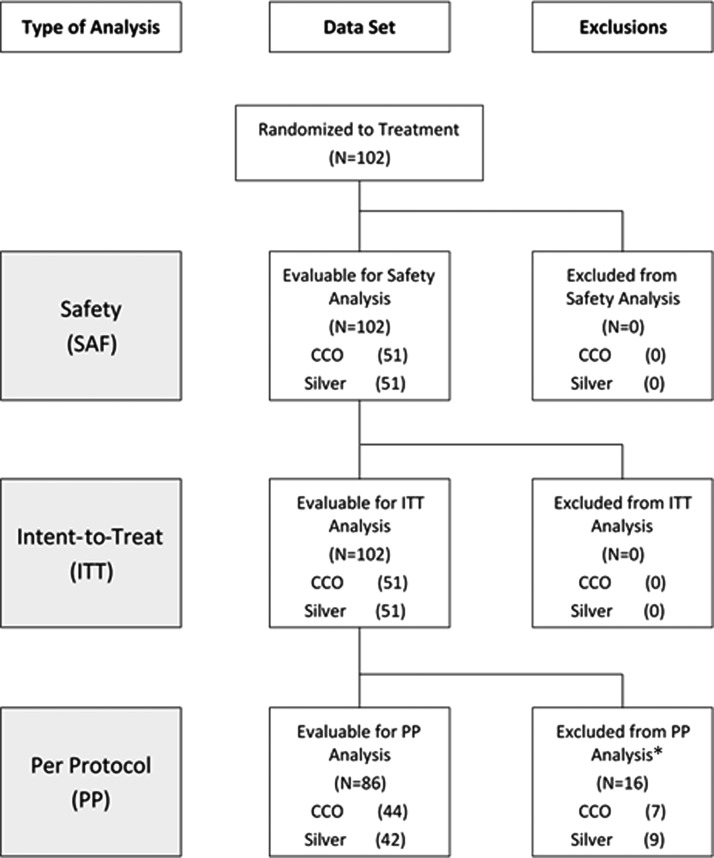
Disposition of study subjects. *Reasons for exclusion from per protocol analysis given in text.

The disposition of enrolled subjects is given in Fig. 1. Overall, 102 subjects were randomized, all of whom received at least 1 study treatment, and 82 completed the study. Twenty subjects were discontinued from the study before completion for the following reasons: 13 for adverse event (including new DFU), 4 for protocol deviations, and 1 each for loss to follow-up, withdrawal of consent, and nonadherence with study treatment. Sixteen subjects were excluded from the PP analysis for the following reasons: 13 for withdrawal from the study and 3 for protocol violations. Fifty-one subjects each were randomized to receive treatment with CCO and silver-containing products; descriptions of the silver-containing products used in the control group of this study are given in Table 1. Demographics and baseline wound characteristics of the ITT/safety population are given in Table 2 and were comparable between groups.

**Table 1.** Investigator-selected silver treatment

*Silver Product*	*N*^a^* = 52*
Ag+ coated nylon in alginate/caboxymethylcellulose	20
Ag_4_O_4_ coated nanoparticles in hydrogel	11
Ag+ in hydrogel	7
Ag+ integrated into caboxymethylcellulose fibers	7
Ag_2_O in hydrolyzed collagen gel	3
Silver sulfadiazene	3
Ag+ impregnated foam dressing	1

^a^ One subject was treated with a combination of a silver foam dressing and a silver collagen gel and is thus counted twice.

**Table 2.** Demographic and baseline wound characteristics of the enrolled subjects

	*All (*n* = 102)*	*Collagenase (*n* = 51)*	*Silver (*n* = 51)*	p
Age, year (mean [SD])	57.0 (12)	56.4 (13.1)	57.6 (10.8)	0.6221
Gender (*n* [%] male)	78 (76.5)	42 (82.4)	36 (70.6)	0.1613
Race (*n* [%])				
White/Caucasian	94 (92.2)	47 (92.2)	47 (92.2)	1.000
Black/African American	6 (5.9)	3 (5.9)	3 (5.9)	
Other	2 (2)	1 (2)	1 (2)	
BMI, kg/m^2^ (mean [SD])	34.0 ± 7.0	34.3 ± 7.7	33.6 ± 6.3	0.5901
Ankle brachial index (mean [SD])	1.0 ± 0.1	1.0 ± 0.1	1.0 0.2	0.4725
Target ulcer area, cm^2^ (mean [SD])	1.6 ± 1.9	1.6 ± 1.8	1.7 ± 2.0	0.6602
Target ulcer location (*n* [%])				
Forefoot	75 (73.5)	33 (64.7)	42 (82.4)	0.1113
Hallux	16 (15.7)	12 (23.5)	4 (7.8)	
Heel	7 (6.9)	3 (5.9)	4 (7.8)	
Medial forefoot	3 (2.9)	2 (3.9)	1 (2.0)	
Lateral heel	1 (1.0)	1 (2.0)	0	
Target ulcer duration, days	112.4 ± 76.3	106.8 ± 75.2	118.1 ± 77.7	0.4591

BMI, body mass index; SD, standard deviation.

**Figure 2.**
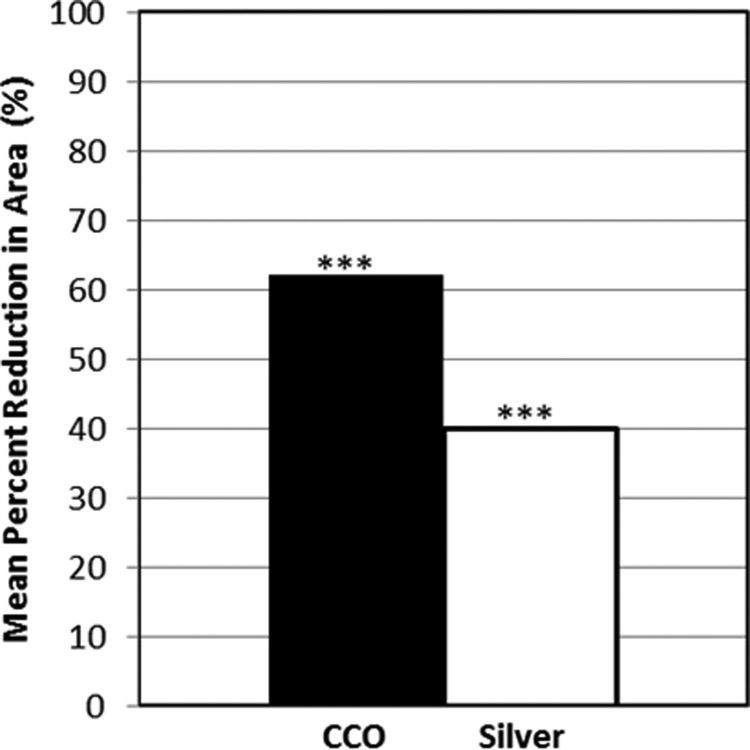
Mean percent reduction in ulcer area from baseline to end of treatment by group in the intent-to-treat population (analysis of covariance). ***p < 0.0001, end of treatment versus baseline, within group *t*-test. CCO, clostridial collagenase ointment.

The baseline mean ulcer area in the CCO group was 1.6 ± 1.8 cm^2^, and by the end of the 6-week treatment period, the mean percent reduction in ulcer area was 62.0% (*p* < 0.0001 for change from baseline). In the silver group, the baseline mean ulcer area was 1.7 ± 2.0 cm^2^ and was reduced by 40.0% (*p* < 0.0001 for change from baseline) by the end of the treatment period (Fig. 2). The difference of 22% (95% confidence interval: −1.9–45.9) between treatment groups, while clinically meaningful in favor of CCO, was not statistically significant (*p* = 0.071). No additional gains in the mean percent reduction in area were observed through the additional 4-week follow-up period in either group. At the end of the study, cumulative mean percent reductions in area were 63.6% and 41.4% in the CCO and silver groups, respectively (*p* = 0.065). Similar results were obtained in the PP dataset at the end of treatment and the end of the study. Debridement was performed 305 times in the CCO group (mean 7.3 debridements per subject) and 272 times (6.8 per subject) in the silver group (not significant).

In the ITT population, the stepwise regression method demonstrated that the percentage reduction in target ulcer area was estimated to be 25.6 (95% CI: [2.733–48.490]) percentage points greater in the collagenase group than in the Silver group (Table 3). This difference was statistically significant (*p* = 0.029). Similar results were obtained in the PP population.

**Table 3.** Percentage reduction in target ulcer area using stepwise regression model

				*95% Confidence Interval for Difference*		
	*CCO Estimate*	*Silver Estimate*	*CCO—Silver*	*Lower*	*Upper*	p
Percentage reduction in area (baseline–visit 7)	22.86	−2.75	25.61	2.733	48.490	0.029

CCO, clostridial collagenase ointment.

For ulcers that closed by the end of the treatment period (CCO group = 11, silver group = 7), the mean time to closure was 31.1 ± 9 days in the CCO group and 37.1 ± 7.7 days in the silver group. By the end of follow-up, the time to closure among closed lesions was 43.0 ± 18.6 and 50.8 ± 14.6 days, respectively. While the results favor the CCO group, they were not statistically significant.

At baseline, all ulcers were colonized by between 1 and 8 different species of bacteria (mean 3.3 ± 1.4). The most commonly sampled species along with the percentage of ulcers closed when the organism was present at baseline are given in Table 4. Only *Staphylococcus epidermidis,* methicillin-resistant *Staphylococcus aureus* (MRSA), and *Pseudomonas aeruginosa* had closure rates below the overall mean of ∼30%. The bacterial bioburden combining all bacterial species ranged from 9.20 × 10^3^ to 9.30 × 10^10^ colony-forming units (CFU)/g among CCO-assigned patients and from 4.50 × 10^4^ to 2.05 × 10^10^ CFU/g in silver-assigned patients. There was no significant association between bacterial bioburden at baseline and wound healing (logistic regression, *p* = 0.813). During the 6-week treatment period, there were numerically more incident target ulcer infections in the silver group (11, 21.6%) than in the collagenase group (5, 9.8%; *p* = 0.208).

**Table 4.** Bacteria most frequently present at baseline and closure rates

*Species*	*N*	*Proportion Closed (%)*
*Staphylococcus aureus*, MSSA	36	41.7
*Proteus mirabilis*	12	41.7
*Corynebacterium striatum*	13	38.5
*Enterococcus faecalis*	34	35.3
*Corynebacterium amycolatum*	10	30
*Streptococcus agalactiae*	21	28.6
*Pseudomonas aeruginosa*	12	25.0
*Staphylococcus epidermidis*	18	22.2
*Staphylococcus aureus* MRSA	16	18.8

MRSA, methicillin-resistance *Staphylococcus aureus*; MSSA, methicillin-sensitive *Staphylococcus aureus*.

In the safety analysis, 70 treatment-emergent adverse events were recorded in 24 subjects (47.1%) of the CCO group and 92 in 29 subjects (56.9%) of the silver group. Of these, only one was deemed treatment related (skin burning sensation in the silver group). There were five serious adverse events in five subjects (9.8%) of the CCO group and nine in eight subjects (15.7%) of the silver group; none was deemed treatment related.

## Discussion

This study demonstrates a 55% relative greater reduction in mean percent area of DFUs treated for 6 weeks with CCO compared to silver-containing products. This difference, while not statistically significant, is clinically relevant. Interestingly, after adjustment for site, ulcer age, ABI, and BMI, the percent decrease in wound area did show statistical significance in favor of CCO. This result underscores the importance covariates and confounding factors can have, particularly in a population that is diverse and may present with numerous comorbidities in addition to diabetes mellitus. Ulcers achieving closure did so ∼1 week faster in the CCO group (although not reaching statistical significance). CCO-treated ulcers also demonstrated a 55% relative reduction in infections compared to silver-containing products. Both treatments were well tolerated, with no CCO treatment-related adverse events observed.

Silver-containing products are believed to convey an antimicrobial benefit mediated by anti-infective activity of silver ions, although a recent evidence-based review found mixed results and thus was not able to conclude that silver-containing wound dressings convey antimicrobial efficacy.^15^ This potential benefit is balanced against the known cytotoxic effects of ionic silver which may impair healing in the ulcer bed.^8,9,16,17^ CCO, on the other hand, provides exogenous proteolytic activity with the potential to maintain a more organized wound as the wound progresses toward closure. Depending on the level of free silver ions, some silver-containing products may inhibit collagenase activity.^14^ Therefore, care must be taken if these two therapies are used concomitantly.

In this study, subjects were considered to be free of ulcer infection when enrolled. During the course of the 6-week treatment period, fewer ulcer infections were observed in subjects treated with clostridial collagenase than subjects treated with a silver-containing product. However, CCO is not indicated for the prevention of infection. It is hypothesized that the smaller number of infections observed in the CCO group in this study was due to the removal of the necrotic tissue from the wound bed, limiting the ability of bacteria to thrive in the wound. Further study will be required to test this hypothesis.

The lack of statistical significance for the primary endpoint of this study warrants consideration. From a technical standpoint, the study was powered assuming an effect size of 0.6 for collagenase and was underpowered for the observed effect size of ∼0.4. Perhaps more importantly, the trend toward statistical significance observed in this study is consistent with previous studies. Milne *et al.* demonstrated more complete debridement of chronic ulcerative wounds with collagenase than standard hydrogel therapy.^18^ Tallis *et al.* reported significantly greater reductions in DFU area and significantly better response rates in collagenase-treated lesions compared to saline-moistened gauze coupled with serial sharp debridement.^13^ Motley *et al.* demonstrated greater reductions in wound area with 6 weeks of collagenase treatment compared to sharp debridement used with good ulcer care, although the observed difference (68% vs. 36%, respectively) was not statistically significant; however, this was a hypothesis-generating rather than hypothesis-testing study, and sample size was selected quasi-arbitrarily rather than based on *a priori* power analysis.^7^ In a *post hoc* analysis of the data from Motley *et al.*, wound infection rates were similar between groups (10.7% for CCO, 11.8% for debridement with good ulcer care, 63% of which included silver-containing products; Motley *et al.*, Unpublished Data^*^). Also, Jimenez *et al.* reported that CCO provided numerically (but not statistically significantly) greater reductions in DFU area compared to hydrogel with up to 12 weeks of therapy; mean percent reductions were numerically greater for CCO than hydrogel at 12 of 12 time points and averaged 55% versus 41%, respectively.^6^ In the latter study, ulcers unresponsive to randomized therapy at 4 weeks were crossed over to the other treatment, after which 33% of ulcers switched to CCO closed versus only 8% switched to hydrogel. Taking these studies as a whole along with the current study, CCO consistently produced numerically—and often statistically—better outcomes than standard therapy in every study.^19^ The current study, considered in combination with the existing body of literature with which the current study's findings are consistent, provides additional evidence to help clarify the optimal management of DFUs. CCO produces more rapid reduction in ulcer area, reduces time to closure in lesions achieving closure, and promotes resolution of inflammation in the wound bed as the ulcer progresses toward closure.

This study's design featured several important features. We utilized an objective protocol for the measurement of lesion size using the ARANZ Silhouette system. This device combines a digital camera and a dual laser platform that accounts for image scale and skin curvature when measuring wound surface area. The device has been shown to be highly reproducible with excellent intra- and interobserver reproducibility.^20^

The current study has some limitations that merit mention. First, there was not a standard silver-containing product for DFU treatment. However, the choice between selecting a “standard care” (one dressing used for all patients randomized to silver) or “standard of care” (Investigator uses dressing he/she would normally select based on patient characteristics and local practice norms) is essentially the choice between intrinsic validity (narrowly defined criteria to reduce variability) and extrinsic validity (generalizable to the “real world”). Neither is wrong, and both have strengths. We chose the latter because we felt that generalizability of our results and translation to clinical practice were a higher priority for the study questions we were asking. As part of the training for the study, investigators were encouraged to use the silver-containing product that they thought was most appropriate for a given ulcer. The assessment of the target ulcer as infected or not infected at the screening visit (and thus study eligibility) was based on the Investigator's judgment of signs and symptoms of infection rather than waiting for culture results and using numeric criteria. This is reasonable as the decision to treat with systemic antibiotics is also often based on visible criteria rather than laboratory results. The most important limitation of this study was its small size. The study was powered to detect a large effect size and was unable to establish the statistical significance of the observed smaller effect size, although a *post hoc* covariate analysis did reach significance.

In summary, DFUs provided with ongoing enzymatic debridement by CCO achieve faster and greater reductions in ulcer area than when treated with silver-containing products with an incidence of ulcer infection at least as low as for silver-containing products.

## Innovation

Clostridial collagenase provides ongoing enzymatic debridement supplemental to intermittent sharp debridement in the management of DFUs. Silver-containing products are often used to confer prophylactic antimicrobial coverage. The clinical impact is that CCO treatment not only results in more rapid progress toward closure than treatment with silver-containing products but also is at least as effective as the silver products in limiting the incidence of ulcer infections.

Key Findings

• The mean percent decrease from baseline in ulcer area over the treatment period was highly significant for both the collagenase (*p* < 0.0001) and for the silver (*p* < 0.0001) treatment groups.
• Ulcers treated with clostridial collagenase had a 55% greater mean reduction in area from baseline when compared with the silver control group at the end of treatment (*p* = 0.071).
• Silver treatment provided no advantage over clostridial collagenase in the incidence of observed ulcer infections during the treatment period.
• Both treatments were found to be safe under the conditions of the study.

## About the Authors

**Travis A. Motley, DPM, MS, FACFAS,** is board certified in foot surgery and reconstructive rearfoot and ankle surgery. He is the Director of the podiatry surgical residency program at John Peter Smith Hospital in Fort Worth, Texas and Professor in the Department of Orthopedic Surgery at John Peter Smith Hospital and Acclaim Physician Group. **Joseph M. Caporusso, DPM, MPH,** is a practicing podiatrist in McAllen Texas, He is also the founder, CEO, and certified principal investigator at Futuro Clinical Trials, a clinical research facility dedicated to the treatment of chronic nonhealing diabetic foot ulcerations and other pedal conditions. Dr. Caporusso holds appointments at the University of Texas Rio Grande Valley medical school and the Western University School of Podiatric Medicine. He has participated in numerous clinical trials as the principal investigator. **Darrell L. Lange, PhD,** is a Senior Manager in Global Clinical Operations at Smith & Nephew. He was the study manager for this study. **Robert Eichelkraut, MBA,** is Vice President of Global Clinical Operations at Smith & Nephew. **David Innes Cargill, PhD,** was Director of Global Clinical Operations at Smith & Nephew and was the medical monitor for the study at the time of study design and execution. **Jaime E. Dickerson, Jr., PhD,** was the Vice President of Medical and Clinical Affairs at Smith & Nephew at the time of study design and execution. He is also Adjunct Associate Professor in the Graduate School of Biomedical Sciences at the University of North Texas Health Science Center, Fort Worth, TX.

## Abbreviations and Acronyms

ABI: ankle-brachial index
ANCOVA: analysis of covariance
BMI: body mass index
CCO: clostridial collagenase ointment
CFU: colony-forming units
CMH: Cochran–Mantel–Haenszel
DFU: diabetic foot ulcers
ITT: intent-to-treat
PP: per protocol
SD: standard deviation

## Appendix 1

### Inclusion Criteria

**Table:**

1. Provide written informed consent, which will consist of reading, signing, and dating the informed consent document after the investigator, subinvestigator, or other designated study staff member has explained the study procedures, risks, and contact information.
2. Eighteen years of age or older, of either sex, and of any race or skin type.
3. Willing and able to make all required study visits.
4. Able to follow instructions and perform the dressing changes at home or have a caregiver who can perform the dressing changes according to the protocol.
5. Willing to use an appropriate off-loading device to keep weight off of foot ulcers.
6. An ulcer present on any part of the plantar surface of the neuropathic foot or hallux, which is 0.5–10 cm^2^ inclusive (as measured at the screening visit using the ARANZ Silhouette imaging device). The target ulcer duration must be ≥6 weeks but not more than 52 weeks (12 months) as documented in the subject's history or by subject report of onset, which requires debridement.
7. Adequate arterial blood flow as evidenced by an ankle-brachial index (ABI) of >0.70 and ≤1.20. If ABI >1.2, perfusion at or near the site of the ulcer should be confirmed; the foot is warm to the touch and has palpable pulses.
8. Separation of at least 5 cm (closest ulcer edge to other closest ulcer edge) if ≥2 ulcers are present as measured using the ARANZ Silhouette imaging device.
9. Diabetes mellitus (Type 1 or 2) requiring insulin or oral/injectable medications to control blood glucose levels.
10. Target ulcer is not infected based on clinical assessment.

### Exclusion Criteria

**Table:**

1. Contraindications or hypersensitivity to the use of clostridial collagenase or products containing silver.
2. Participation in another clinical trial within 30 days of Visit 1, or planned participation overlapping with this study.
3. Bleeding disorder that would preclude sharp debridement during the study.
4. Active cellulitis of the target ulcer, lymphangitic streaking, deep tissue abscess, gangrene, or infection of muscle, tendon, joint, or bone.
5. Infection with systemic toxicity or metabolic instability (*e.g.*, fever, chills, tachycardia, hypotension, confusion, vomiting, leukocytosis, acidosis, severe hyperglycemia, azotemia).
6. A target ulcer which involves the underlying tissues of tendon, muscle, or bone.
7. Diagnosis of chronic granulomatous disease, leukocyte adhesion defects, or severe neutropenia.
8. Current treatment (at the time of the screening visit) with any of the following:
• Systemic corticosteroids. If corticosteroid treatment was for ≥10 days, there must be a 1 week interval between discontinuation and screening.
• Immunosuppressive agents
• Chemotherapeutic agents
• Antiviral agents
• Systemic antibiotic therapy (for any reason) or topical antibiotic treatment of the target ulcer
9. Treatment of target ulcer with bioactive therapies within 1 month of screening:
• Platelet-derived growth factor (*e.g.*, Regranex^®^)
• Living skin equivalent (*e.g.*, Apligraf^®^)
• Dermal substitute (*e.g.*, Dermagraft^®^, Integra^®^, Oasis^®^, etc.)
• Amniotic membrane products (*e.g.*, EpiFix^®^, Grafix^®^, etc.)
10. Prior treatment of target ulcer for any length of time with clostridial collagenase ointment (SANTYL^®^).
11. Radiation therapy to the target lower extremity within 30 days before screening.
12. Medical or physical condition that, in the opinion of the investigator, would preclude safe subject participation in the study.
13. Blood counts and blood chemistry values as follows:
• Alanine aminotransferase >3 × upper limit of normal
• Aspartate aminotransferase >3 × upper limit of normal
• Gamma glutamyl transferase >2.5 × upper limit of normal
• Serum albumin <2.0 g/dL
• Alkaline phosphatase >500 U/L
• Serum blood urea nitrogen >75 mg/dL
• HbA1c >12%
• White blood cells <2.0 × 10^9^/L
• Platelet count <50 × 10^9^/L
• Prealbumin levels of <10 mg/dL
• Serum total bilirubin >3.0 mg/dL
• Serum creatinine >4.5 mg/dL
• Hemoglobin <8.0 g/dL
• Absolute neutrophil count <1.0 × 10^9^/L

### Additional Exclusion Criteria Before Randomization

**Table:**

1. Use of excluded concomitant medications or therapies between Screening and Visit 1.
2. A clinically diagnosed infection of the target ulcer requiring treatment.
3. Muscle, tendon, or bone exposure in the target ulcer.
4. After debridement at Visit 1, the ulcer area is <0.5 cm^2^ or >12 cm^2^ (as measured at the using the ARANZ Silhouette imaging device).

## Notes

^*^ Sharp surgical debridement with and without CCO, Smith & Nephew, 2013.
